# Five Things Not to Do When Discovering a Biosecurity Vulnerability

**DOI:** 10.1089/apb.2023.0038

**Published:** 2024-09-18

**Authors:** Piers D. Millett

**Affiliations:** International Biosecurity and Biosafety Initiative for Science (IBBIS), Washington, DC, USA.

**Keywords:** behavior, biosecurity, genetic, synthetic DNA, risk assessment, engineering

## Abstract

**Introduction:**

Tools to screen orders submitted to companies synthesizing nucleic acids or machines that can synthesize them are vital to help ensure the building blocks for pathogens, toxins, or other biotechnology that could cause harm are kept in the right hands.

**Methods:**

The author has argued that it would be worrying if someone discovering a vulnerability in a nucleic acid synthesis tool was to (1) publicly release it without a “patch,” (2) set an impossible timeframe to patch it before public release, (3) refuse to report it without a reward, (4) test it on a tool without the developer's/operator's consent, or (5) create a real biological hazard while exploring hypothetical biosecurity vulnerabilities.

**Conclusion:**

It will be a much harder challenge to develop a parallel set of behaviors desirable in such circumstances. This is a process that should start now. There will soon be a need for a safe, balanced, and reliable reporting infrastructure.

Many of the articles in this special edition discuss tools to screen orders submitted to companies synthesizing nucleic acids or machines that can synthesize them. Such tools are vital to help ensure the building blocks for pathogens, toxins, or other biotechnology that could cause harm are kept in the right hands. Preventing malicious actors from acquiring or using these key biological materials is an important access control that enables us to maximize the benefits from biotechnology while minimizing the risks. Next steps for strengthening these tools and the arrangements that contain them have been explored previously.^1,2^

The tools used to screen synthetic nucleic acid orders are normally software.^†^ As with other software, there will be potential vulnerabilities that enable unintended use or circumvention of security controls. For nucleic acid synthesis screening software, vulnerabilities potentially allow an irresponsible or malign actor to acquire genetic material that would otherwise be not readily attainable.

There are well-established good practices when developing and testing such software, including how to secure the software itself. Others have already pointed out that there are similarities between biosecurity and cybersecurity. Indeed, the creation of cyberbiosecurity as an independent field of study is intrinsic recognition that there are shared threats and risk mitigation approaches.^3^ The overlap between artificial intelligence and biotechnology will likely lead to biosecurity and cybersecurity becoming increasingly interwoven. There have been calls to learn lessons from cybersecurity and apply them to biosecurity. For example, to put the hackers to work to “Invite white hat hackers to do their best to defeat your system. Better yet, hire some hackers of your own. It's best to have those who find breaking into a security system an irresistible challenge on your team.”^4^

Efforts are already underway to adapt common cybersecurity approaches for use in biosecurity. For example, the recent White House Executive Order on the Safe, Secure, and Trustworthy Development and Use of Artificial Intelligence requires the U.S. Government to develop frameworks “to conduct structured evaluation and stress testing of nucleic acid synthesis.”^5^ This effectively requires creating a framework for penetration testing and vulnerability disclosure. Importantly, this assessment cannot stop at discovering and disclosing vulnerabilities in the software. It will require discovering and disclosing holes that would allow a malicious actor to illicitly acquire nucleic acids that could be used to cause harm—straying from traditional cybersecurity firmly into biosecurity.

There are important differences between cybersecurity and biosecurity. For example:

“Digital vulnerabilities have a shorter half-life than biological threats. Measures to promote disclosures and crowd-sourced problem-solving will therefore have a larger immediate impact on cybersecurity. On the other hand, reporting “vulnerabilities” in the bio realm poses a greater security risk when countermeasures are not and may never be available.”^6^

At the present time, there is also a difference in the distribution of the user communities. For example, gene synthesis capabilities are currently largely centralized in a limited number of commercial suppliers. In contrast, the users of traditional IT infrastructure are highly decentralized. This *status quo* could change in the near future as more desktop gene printers become commercially available.^7^

What will happen when someone discovers a vulnerability in a screening tool? What happens if that person is an independent third party? Who should they tell, how and when? What should happen with that information? Who gets to decide? These are tricky questions. Creating a safe, balanced, and reliable reporting infrastructure will take time, work, and resources.

It is hard to say what should happen. It is much easier to think about what should not. This may be particularly important in the short-term as vulnerability reporting is a classic example of the Unilaterist's Curse—where a single actor has the power to produce an outcome undesirable to the rest of their community.^8^ Our collective security would suffer if someone found a vulnerability and they:

## Publicly Release a Vulnerability Without a “Patch”

It may not be possible to patch a vulnerability. As a result, announcing a hole in the current screening system to the world, without there being a fix, would seem to aid those that desire using biotechnologies and undermine the efforts to prevent that from happening. There are powerful incentives in the cybersecurity world to make such announcements, and they are common at major hacking conferences around the world. What will happen if this is applied to nucleic acid synthesis screening?

The cost of an unpatched hole in nucleic acid synthesis screening could have serious consequences. Based on the impact of the COVID-19 pandemic, it could be argued that the harm caused by a self-replicating biological virus may be on a different scale than that potentially caused by a computer virus. Unlike in cybersecurity, we do not have a choice of operating system or computer system. We may not have options to deal with a biological exploit.

Vulnerabilities should be reported. It is critical that holes in the system are discussed with those that need to know. That could include those making the tools, as well as national and international policy makers and biosecurity professionals. Disclosing vulnerabilities need not be binary—how and to whom they are communicated is important.

## Set Impossible Timeframes

It is common in cybersecurity for someone discovering a vulnerability to report it to the tool manufacturer or a trusted intermediary and then start a countdown toward public release. For example, the U.S. Cybersecurity and Infrastructure Security Agency “may disclose vulnerabilities as early as 45 days after the initial attempt to contact the vendor is made regardless of the availability of a patch or update.”^9^

For nucleic acid synthesis screening, the use of a countdown to public release might be useful if the vulnerability is a traditional cybersecurity issue in the software. It would be necessary that a suitable patch could be developed and distributed within the time frame. If the vulnerability is about biosecurity—that is, would potentially enable a bad actor to circumvent screening or access nucleic acid synthesis that they would otherwise not be able to access, the use of a countdown to public release may not be appropriate. The vulnerability may not be patchable at all. Alternatively, it may not be patchable within the timeframes traditionally associated with cybersecurity. More consideration is warranted as to if countdown to public release is useful in this context and, if so, what are the suitable timeframes.

## Refuse to Disclose a Vulnerability Without a Reward

Another common cybersecurity practice involves bug bounty platforms.^10^ These are run by software companies and tool developers, or as independent third-party services. They often offer rewards for reporting vulnerabilities. For example, Google offers rewards over $31,000 for reporting some vulnerabilities.^11^

What a “bug” looks like in this context is less clear than for traditional cybersecurity. For example, is it a “bug” if a vulnerability enables an order to be made for a potentially hazardous but not controlled gene? Is it a “bug” if a vulnerability enables an order to be made for a gene (or gene's product) that requires heavy chemical modification before it is hazardous?

While there is the potential for biosecurity tool developers to offer rewards for reporting vulnerabilities, they often have many orders of magnitude fewer resources than large software companies. There may be few rewards for reporting a critical biosecurity vulnerability beyond contributing to global biosecurity and helping to build important norms in the industry offering synthetic nucleic acids.

## Conduct Unauthorized Testing of the Vulnerability

There have been examples of third-party biosecurity penetration testing on nucleic acid synthesis screening that has been done with the consent of the companies making or operating the screening tools.^12^

Penetration testing with prior consent enables verification of the intent of the tester. It allows suitable measures to be put in place to protect the interests of both those doing the testing, and those being tested, such as nondisclosure agreements. It helps ensure that those doing the testing are protected. If the tool user does not know that someone is attempting to bypass their system as part of a benign effort to enhance security, it could be mistaken for a malicious act. It can also enable the involvement of law enforcement or other government actors, which reduces the chance of the tester being identified as a malicious actor and also helps to ensure those enforcing biosecurity are up to speed with possible vulnerabilities. This in turn helps build more responsive and effective national rules and oversight measures.

While it is true that few security cybersecurity researchers are prosecuted for their efforts, the same may not hold true for biosecurity. The laws on misusing computers are very different from those preventing the proliferation of weapons of mass destruction. Someone that has identified a potential vulnerability should not attempt to test it on a nucleic acid synthesis-screening tool without the consent of the tool maker and/or operator.

## Turn the Vulnerability into Biology

There is a big difference between discovering a potential vulnerability in nucleic acid synthesis screening tools and actually making something that could cause harm. In general, in silico vulnerabilities should not be translated into *in vivo* hazards. It could be a criminal offense to actually acquire a dangerous biological agent, or a part of one such as a controlled gene, or sequence encoding a toxin—even if it is done with the intent of improving biosecurity tools. Demonstrating in practice, rather than positing a theory, that a vulnerability could result in a biological threat may compound information hazards being generated—where the spread of knowledge itself changes the threat environment (rather than the proliferation of a material, for example).^13^ In general, to highlight a potential concern, the minimal number of steps toward an actual biological hazard should be taken. Any movement into biology should probably be done by threat assessment professionals inside a suitably robust security classification framework and may need to involve steps to reduce the hazard, such as through the use of attenuations.

## Conclusion

This commentary has explored undesirable behaviors. It is a necessary, but much harder challenge to develop a parallel set of desirable behaviors. Determining what a vulnerability reporting process for nucleic acid synthesis screening should involve will require a structured and sustained process that involves and is seen as legitimate by the cybersecurity and biosecurity communities as well as those developing and using these screening tools. Such a process could also explore vulnerability reporting for customer screening as well as order screening.

There will be important lessons to be learned from traditional cybersecurity, such as the use of bug bounty platforms. It will also be vital to consider how cybersecurity approaches may need to be adapted for use with biology, for example, re-examining timeframes for resolving vulnerabilities before making them publicly available. Such efforts must also be forward looking, for example, addressing both software vulnerabilities that allow acquisition of known threats as well as software vulnerabilities that allow acquisition of novel threats. It is to be hoped that as *desirable* behaviors and activities are explored, *undesirable* behaviors and activities are not inadvertently endorsed.

## Notes

^†^
Customer screening, another important aspect of safeguarding nucleic acid synthesis, is less frequently software based and is outside the scope of these remarks.
